# A long non-coding RNA LINC00461-dependent mechanism underlying breast cancer invasion and migration via the miR-144-3p/KPNA2 axis

**DOI:** 10.1186/s12935-020-01221-y

**Published:** 2020-04-26

**Authors:** Qiang Zhang, Xiaoyan Jin, Wenbiao Shi, Xin Chen, Wenyang Pang, Xiaodong Yu, Linjun Yang

**Affiliations:** Department of Breast Central, Taizhou Municipal Hospital, No.138 Zhongshan Road, Taizhou, 318000 Zhejiang China

**Keywords:** LINC00461, KPNA2, miR-144-3p, Breast cancer, Invasion, Migration

## Abstract

**Background:**

The purpose of this study was to explore the regulatory mechanism of the long non-coding RNA (lncRNA) LINC00461 underlying the breast cancer invasion and migration via the miR-144-3p/KPNA2 axis.

**Methods:**

Bioinformatics methods were applied to screen differentially expressed mRNAs, miRNAs and lncRNAs for construction of a competing endogenous RNA (ceRNA) network. LINC00461, KPNA2 and miR-144-3p were identified, and KPNA2 was predicted to be a target of miR-144-3p and significantly correlated with breast cancer prognosis. To make the findings more convincible, we used qRT-PCR to detect the expression levels of LINC00461 and miR-144-3p in breast cancer cells, and conducted western blot to determine KPNA2 protein level. Then, RIP was performed to assess the combination between miR-144-3p and LINC00461 or KPNA2, and dual-luciferase reporter assay was used to validate the targeted relationship between miR-144-3p and KPNA2. Furthermore, Transwell was employed for the examination of cell invasion and migration in breast cancer.

**Results:**

LINC00461 was predicted to regulate KPNA2 through sponging miR-144-3p as revealed by the ceRNA network. Besides, LINC00461 and KPNA2 were found to be remarkably highly-expressed in breast cancer cells, while miR-144-3p was poorly-expressed. Silencing LINC00461 could promote miR-144-3p expression, thus inhibiting cell invasion and migration. In addition, KPNA2 was confirmed to be a direct target of miR-144-3p. Silencing miR-144-3p or overexpressing KPNA2 could reverse the inhibitory effect of LINC00461 silencing on cell invasion and migration in breast cancer.

**Conclusion:**

LINC00461 promoted the expression of KPNA2 by competitively binding to miR-144-3p, thereby promoting the invasion and migration of breast cancer cells.

## Background

Breast cancer is the most common disease in women and occurs globally. Generally, breast cancer is predicted to take 25% of all cancer types and 15% among all cancer-related deaths, whereas those in the developed countries are much higher as approximately 50% and 38%, respectively. In China, breast cancer is the main cause leading to cancer death in women, with nearly 268,600 confirmed cases in 2015 [1]. Due to the heterogeneity [2], investigating the mechanisms underlying the occurrence and development of breast cancer is of great importance for identifying novel sensitive and effective therapeutic targets.

Long non-coding RNAs (lncRNAs) are RNA transcripts harboring over 200 nucleotides [3]. Much effort has suggested that lncRNAs are relevant to the pathogenesis of breast cancer [4, 5]. For example, LINC00978 exhibits up-regulated expression in breast cancer tissues and cells and can be used as a potential biomarker for predicting prognosis [6]. In addition, LINC00617 as a crucial regulator involved in epithelial–mesenchymal transition (EMT) process can positively function on tumor progression and metastasis via activating Sox2 transcription, which makes it possible for LINC00617 severing as a potential therapeutic target in breast cancer [7]. Furthermore, LINC00628 has been found to be decreased in breast cancer, and its overexpression is able to suppress cell proliferation, invasion and migration, also can accelerate cell apoptosis that is involved in regulation of Bcl-2/Bax/Caspase-3 signaling pathways [8].

LINC00461 is a novel cancer-related lncRNA generated by transcription of the gene in the intergenic region on human chromosome 5 [9]. Several studies have described that LINC00461 is a vital player in glioma progression responsible for mediating cell proliferation, invasion and migration via MAPK/ERK, PI3K/AKT and other possible signaling pathways [9], and it can be involved in the LINC00461/miR-149-5p/LRIG2 axis in turn participating in the progression of hepatocellular carcinoma [10]. However, the role of LINC00461 in breast cancer is poorly reported [11].

It has been intensively suggested that lncRNAs are implicated in the occurrence and development of various cancers by serving as competing endogenous RNAs (ceRNAs) [12, 13]. LINC00673, for instance, can sponge miR-150-5p to regulate cell proliferation, migration, invasion and EMT process in non-small cell lung cancer (NSCLC) [14]. LINC00460 can induce the occurrence of nasopharyngeal cancer via the miR-149-5p/IL6 axis [15]. In our study, LINC00461 was discovered to be considerably elevated in breast cancer via bioinformatics analysis, and survival analysis revealed that there was an intimate correlation between KPNA2 and prognosis. Besides, we established a ceRNA network and predicted that LINC00461 might sponge miR-144-3p to target KPNA2, thereby making impact on cell invasion and migration. Our study opens a door for the further research on breast cancer pathogenesis and for the exploration of candidate diagnostic biomarkers as well as therapeutic targets.

## Materials and methods

### Bioinformatics analysis

TCGA database (https://portal.gdc.cancer.gov/) was used to access the expression quantification data of all genes and miRNAs in breast cancer, and “edgeR” package was applied for screening differentially expressed genes, lncRNAs and miRNAs (DEGs, DElncRNAs and DEmiRNAs), with the threshold set as |logFC| > 2 and *p*.adj < 0.05. mircode database (http://www.mircode.org/) was employed to find the potential pairs of DElncRNA-DEmiRNA, whereas miRDB (http://mirdb.org/miRDB/index.html), miRTarBase (http://mirtarbase.mbc.nctu.edu.tw/php/index.php) and TargetScan (http://www.targetscan.org/vert_71/) three databases were used for predicting the target genes of the identified DEmiRNAs. A ceRNA network was established according to the candidate lncRNAs, miRNAs and mRNAs.

### Sample collection

A total of 84 pairs of breast cancer surgery resected tissue samples and matched adjacent normal tissue samples from May 2016 to May 2019 were collected in Taizhou Municipal Hospital, with available detailed corresponding clinical information. All samples were histopathologically diagnosed by an experienced pathologist and cryopreserved in 80 °C. All patients were females aged (26–68) years old with the median age of 53, and they received no radiotherapy or chemotherapy before mastectomy or lumpectomy. This study was performed with the approval of the Ethnic Committee in Taizhou Municipal Hospital, and had obtained the informed consent from all subjects.

### Fluorescence in situ hybridization (FISH)

Paraffin-embedded dewaxed sections were firstly treated with proteinase K (Roche), followed by hybridization using a 300 ng/mL probe (sequence: CAGGACGCTCCGATTCCCACGC; Sigma, Shanghai, China) at 37 °C for 16 h. Then, the sections were exposed to 100 μL of diluted DAPI solution for nucleus staining. Images were captured under a fluorescence microscope and emerged using the Image J software.

### Cell culture and transfection

Human normal breast epithelial cell line MCF 10A (CBP60419) and breast cancer cell lines AU565 (CBP60353), MCF-7 (CBP60380), MDA-MB-157 (CBP60381) and MDA-MB-231 (CBP60382) were all purchased from Cobioer (Nanjing, China). Plasmids including si-NC, si-LINC00461, inhibitor NC, miR-144-3p inhibitor, mimic NC, miR-144-3p mimic, oe-NC, oe-KPNA2 were ordered from GenePharma (Shanghai, China), and the siRNAs were sequenced as below: si-LINC00461: 5′-CTGCAAAGAAGCATAAAATGA-3′; si-NC: 5′-TTCTCCGAACGTG TCACGT-3′.

Before transfection, cells were grown in 6-well plates (3 × 10^5^ cells/well) until the density reached 50%. 250 μL of serum-free Opti-MEM (51985042, Gibco, Gaitherburg, MD, USA) was used to dilute the target plasmids (4 μg) and Lipofectamin 2000 reagent (10 μL; 11668-019, Invitrogen, NY, California, USA), respectively. Thereafter, the dilutions were mixed, and then dripped into cells after 20 min. All cells were maintained in 5% CO_2_ at 37 °C for 6 h, and cultured for additional 36–48 h with fresh mediums.

### qRT-PCR

Total RNA was isolated from tissues and cells using the Trizol (16096020; Thermo Fisher Scientific, NY, USA). A measure of 5 µg of RNA samples were taken for cDNA template synthesis with the cDNA assay kit (K1622; Fermentas Inc., Ontario, CA, USA). miR-144-3p was quantitated by TaqMan MicroRNA Assay (Applied Biosystems, USA) according to the manufacturer’s protocol and qRT-PCR was run on the TaqMan^®^ Universal PCR Master Mix (Applied Biosystems, Foster City, CA). The reaction was designed as below: 5 min of predenaturation at 95 °C, 30 cycles of 40 s at 95 °C, 40 s at 57 °C and 40 s at 72 °C, followed by 10 min of extension at 72 °C. U6 was applied as an endogenous regulator.

LINC00461 and KPNA2 expression levels were determined by qRT-PCR per the TaqMan Gene Expression Assays protocol (Applied Biosystems, Foster City, CA, USA). GAPDH was taken as internal reference. The procedures were shown as 95 °C for 10 min, 35 cycles of 95 °C for 15 s, 60 °C for 30 s and 72 °C for 45 s. The primers used were listed in Table 1. 2^−ΔΔCt^ method was used for the normalization of gene expression levels, where ΔΔCt = ΔCt_(experiment)_ − ΔCt_(control)_, and ΔCt = Ct_(target gene)_ − Ct _(internal reference)_ [16].

**Table 1:** Primer sequence

Gene	Sequence
LINC00461	F:5′-GACATTTACGCCACAACCCACG -3′
	R:5′-AGACAGACCCTCAGATTCCCCA-3′
GAPDH	F:5′-GGGAGCCAAAAGGGTCAT-3′
	R:5′-GAGTCCTTCCACGATACCAA-3′
miR-144-3p	F:5′-GCGCGCTACAGTATAGATGATG-3′
	R:5′-GCTGTCAACGATACGCTACG-3′
U6	F:5′-GGAGCGAGATCCCTCCAAAAT-3′
	R:5′-GGCTGTTGTCATACTTCTCATGG-3′
KPNA2	F:5′- CGAGTCTGCACCTATTTTGGC-3′
	R:5′-GGGCAGCTGGTGTATTAGCA-3′

### Western blot

Total proteins were extracted using the radioimmunoprecipitation assay lysis buffer (RIPA; R0010, solarbio) containing phenylmethanesulfonyl fluoride (PMSF), and the concentration was determined with a BCA protein assay kit (Thermo Fisher Scientific, NY, USA). 30 μg of total protein samples were subjected to polyacrylamide gel electrophoresis (PAGE) at 80 V for 35 min followed by 120 V for 45 min, then transferred into the polyvinylidene fluoride (PVDF) membranes (Amersham, USA). After being blocked in 5% skim milk for 1 h, the membranes were incubated with primary rabbit polyclonal antibodies overnight at 4 °C, then exposed to horseradish peroxidase (HRP)-labeled secondary antibody goat anti-rabbit IgG H&L (ab6721, 1:3000, abcam, Cambridge, UK) at room temperature for 1 h. PBST buffer (PBS buffer supplemented with 0.1% Tween-20) was used to wash the membranes three times after each reaction. The primary antibodies included KPNA2 (ab84440, 1:1000, abcam, Cambridge, UK), E-cadherin (ab15148, 1:500, abcam, Cambridge, UK), N-cadherin (ab76057, 1:1000, abcam, Cambridge, UK), MMP-2 (ab37150, 1:1000, abcam, Cambridge, UK), MMP-9 (ab73734, 1:1000, abcam, Cambridge, UK) and GAPDH (ab9485, 1:2500, abcam, Cambridge, UK). Images were captured under an optical luminometer (GE, USA), and the protein bands were analyzed using the Image Pro Plus 6.0 software (Media Cybernetics, USA).

### Transwell

Tranwell assay was performed as previously described [17]. For migration assay, transfected cells were firstly suspended by serum-free mediums for 24 h. On the following day, cells were digested, centrifuged and resuspended into a final concentration of 2 × 10^5^ cells/mL. 0.2 mL of suspension was planted into upper chambers, and 700 μL of pre-cooled 10% fetal bovine serum (FBS, Gibco, NY, USA)-supplemented Dulbecco’s Modified Eagle Medium (DMEM) was added in the lower chambers. All cells were nurtured in an incubator in 5% CO_2_ at 37 °C. After 24 h of culture, cells still in the upper chambers were softly wiped off with a cotton swab, whereas cells migrated to the lower chambers were treated with methanol within 30 min, and then stained by 0.1% crystal violet for 20 min. Stained cells were observed under an inverted microscope, and 5 randomized fields of the view were selected for cell count.

For invasion assay, cells were prepared to cell suspension (2.5 × 10^5^ cells/mL) and then seeded into the upper chambers that were pre-coated with hydrated extracellular matrix gel (ECM). The lower chambers were filled with 700 μL of DMEM containing 10% FBS. The following procedures were as similar as the above migration assay.

### RNA binding protein immunoprecipitation (RIP)

The combination of miR-144-3p with LINC00461 or KPNA2 was assessed by RIP assay kit (Millipore, USA), and the procedures were taken as suggested by Liu et al. [18]. Cells were firstly washed with pre-cooled phosphate-buffered saline (PBS), and then lysed using the lysis buffer (P0013B; Beyotime) of equal volume on ice for 5 min, after which the protein samples were subsequently centrifuged at 14,000 rpm at 4 °C for 10 min. The supernatant was injected, and the cell extracts were divided into two parts with one part as Input and the other part used for coprecipitation with antibodies. In each coprecipitation system, 50 μL of magnetic beads were resuspended by 100 μL of RIP Wash Buffer, and then incubated with 5 μg of corresponding antibodies according to the groups. Thereafter, the magnetic bead-antibody complexes were washed and then resuspended in 900 μL of RIP Wash Buffer, followed by incubation with 100 μL of cell extracts overnight at 4 °C. Purified complexes were collected on a magnetic base. The antibodies were composed of Ago2 (ab32381, 1:50, abcam, UK) and IgG (control; ab109489, 1:100, abcam, UK). Afterwards, the complexes and the Input were treated by protease K for subsequent PCR analysis.

### Dual-luciferase reporter gene assay

Dual-luciferase reporter gene assay was done for verification of the targeted relationship between miR-144-3p and KPNA2 with the specific steps taken as described by Wang et al. [19] The full length of the KPNA2 3′-UTR was amplified under the PCR reaction. Then the products were digested with restriction enzymes SpeI and HindIII, and cloned into luciferase pmirGLO (Promega, WI, USA) vector, contributing to the generation of wild type KPNA2 vector (KPNA2-WT). Target prediction database was applied for predicting the potential binding sites of miR-144-3p on KPNA2, and the mutant KPNA2 vector (KPNA2-MUT) was established using the PCR site-directed mutagenesis method. Subsequently, the luciferase vectors were co-transfected with either miR-144-3p mimic or mimic NC into HEK-293T cells, with the Renilla luciferase vector pRL-TK (TaKaRa, Dalian, China) applied as internal reference. Dual-Luciferase Reporter Assay System (Promega, Madison, WI, USA) was employed for the determination of luciferase activity in each group.

### Statistical analysis

SPSS 21.0 software (SPSS, Inc, Chicago, IL, USA) was applied for all data processing. The form of mean ± standard deviation (SD) was used for expressing measurement data. Student’s *t* test and one-way analysis of variance (ANOVA) were respectively performed to analyze the comparisons between two groups and among multiple groups. A Chi-square test was employed for analyzing the count data. All results were representative of at least three repeated experiments. Statistical significance was determined with a threshold of *p *< 0.05.

## Results

### Bioinformatics analysis results

**Fig. 1:**
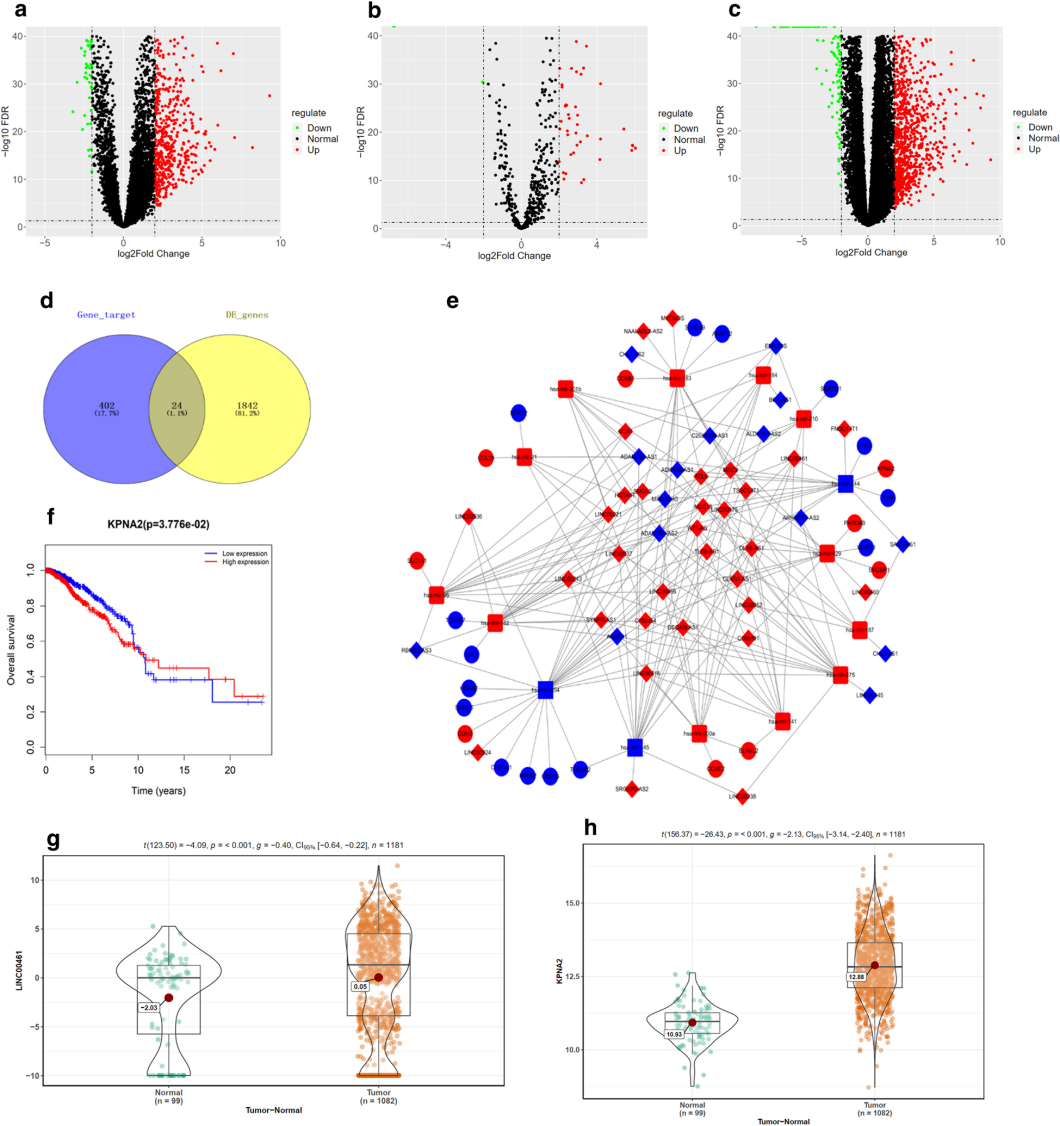
Bioinformatics analysis results. Differential analysis was performed on the gene profiles obtained from the TCGA-BRCA dataset, and eventually **a** 729 DElncRNAs, **b** 67 DEmiRNAs and **c** 1866 DEmRNAs were screened as shown in Volcano Plots. **d** Venn diagram was made to identify the potential target mRNAs among the predicted mRNAs and the 1866 DEmRNAs. **e** A ceRNA network based on the candidate lncRNAs (diamond), miRNAs (square) and mRNAs (circular) was established, with red representing up-regulated genes and blue representing down-regulated genes. **f** Survival analysis was performed on the KPNA2 in the TCGA-BRCA dataset. The abscissa refers to time (years), the vertical coordinate refers to overall survival rate, red line refers to high expression and blue line refers to low expression. Each node refers to a death event. Finally, the expression levels of **g** LINC00461 and **h** KPNA2 were detected in clinical cancer tissue samples and paired adjacent normal tissue samples

In all, 729 DElncRNAs (Fig. 1a) and 67 DEmiRNAs (Fig. 1b) were screened using the “edgeR” package, and mircode database analysis discovered that there were potential binding sites among 46 lncRNAs and 15 miRNAs. TargetScan, miRTarBase and miRDB databases were applied to predict the target genes (n = 426) of the 15 miRNAs, which were then intersected with the 1866 DEmRNAs obtained from the TCGA-BRCA dataset (Fig. 1c). Eventually, 24 DEmRNAs were identified as shown in Fig. 1d. Based on the candidate lncRNAs, miRNAs and mRNAs, a ceRNA network was correspondingly established (Fig. 1e). Among all the DElncRNAs, LINC00461 was found to be considerably increased in breast cancer (Fig. 1g). It has been reported that LINC00461 is involved in the ceRNA network regulation, and responsible for mediating cell proliferation and migration in cancer. Besides, LINC00461 was identified to targeted bind with 7 miRNAs according to the ceRNA network, including miR-210, miR-144, miR-141, miR200a, miR-145, miR-204 and miR-96. Therein, miR-145, miR-204 and miR-144 exhibited a significantly decreased level in cancer cases (Fig. 1h), and survival analysis suggested that the target gene KPNA2 of miR-144 was tightly associated with prognosis of patients with breast cancer (Fig. 1f). Therefore, we reasoned that LINC00461 regulated cell migration and invasion in breast cancer probably via inhibiting miR-144 to promote KPNA2 expression.

### LINC00461 is highly expressed in breast cancer tissues and cells

**Fig. 2:**
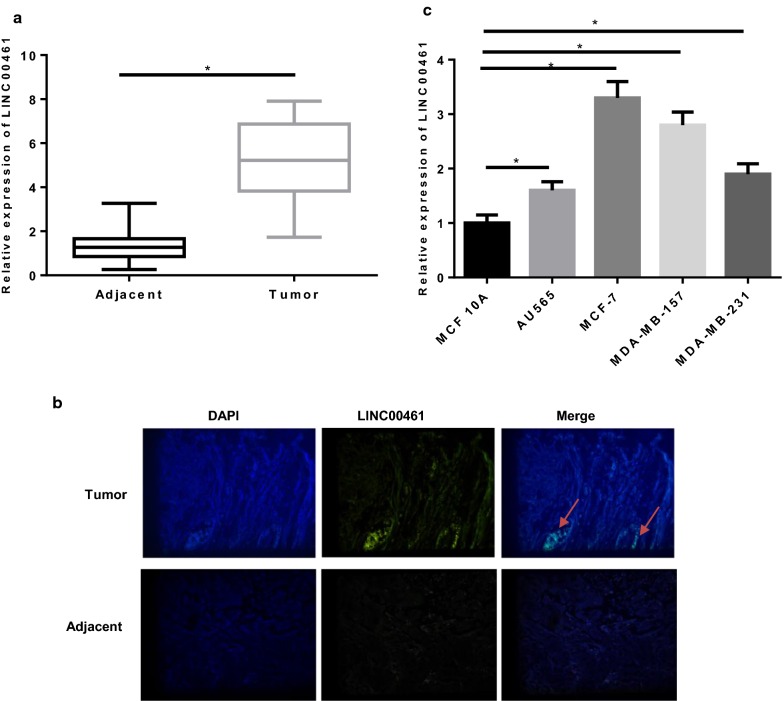
LINC00461 is highly expressed in breast cancer tissues and cells. LINC00461 level was determined in **a** 84 pairs of breast cancer tissue samples and adjacent normal tissue samples via qRT-PCR, and **b** FISH assay for determination of LINC00461 location in breast tissues was performed (the arrow points to the location). **c** LINC00461 was further detected in breast cancer cell lines and normal breast epithelial cell line (*p < 0.05)

qRT-PCR was conducted on 84 pairs of breast cancer tissue samples and adjacent normal tissue samples and found that the expression of LINC00461 was much higher in breast cancer tissues (Fig. 2a). Meanwhile, FISH assay revealed the same result and showed that LINC00461 was mainly located in cytoplasm (Fig. 2b). Then, all patients were classified into the high and low expression groups, with the cutoff value set as the median level of LINC00461 in 84 cancer tissue samples. Combined with the corresponding clinical information, we found that LINC00461 level was intimately correlated with TNM stage, distant metastasis and tumor size, but was independent with age and pathological types (Table 2).

**Table 2:** Correlation between LINC00461 level and clinicopathological features of patients with breast cancer

Clinicopathological features	Cases (n)	LINC00461 expression		*p* value
		Low (n = 42)	High (n = 42)	
Age				0.827
Pre-menopause	38	20	18	
Post-menopause	46	22	24	
Pathological types				0.662
Infiltrating ductal carcinoma	43	23	20	
Infiltrating lobular carcinoma	41	19	22	
TNM stage				0.024
I–II	62	36	26	
III	22	6	16	
Distant metastasis				0.021
Yes	29	9	20	
No	55	33	22	
Tumor size (cm)				0.008
≤ 2	41	27	14	
> 2	43	15	28	

To be more precisely, we examined the level of LINC00461 in one normal breast epithelial cell line MCF 10A and four breast cancer cell lines AU565, MCF-7, MDA-MB-157 and MDA-MB-231. As shown in Fig. 2c, LINC00461 exhibited a remarkably elevated expression in cancer cells in comparison with the normal cells (*p *< 0.05). MCF-7 and MDA-MB-157 cells with relative higher LINC00461 expression were selected for follow up experiments.

### LINC00461 silencing inhibits cell migration and invasion in breast cancer

**Fig. 3:**
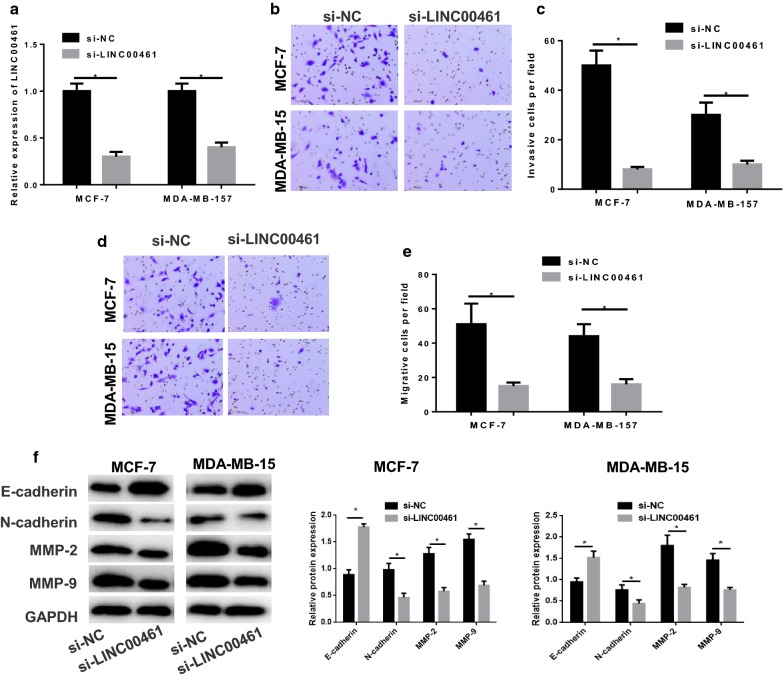
Silencing LINC00461 inhibits cell invasion and migration in breast cancer. si-NC and si-LINC00461 were transfected into MCF-7 and MDA-MB-157 cells. **a** qRT-PCR was performed to detect the transfection efficiency. Then the cells were harvested for **b**, **c** invasion and **d**, **e** migration assessment via Transwell. **f** Western blot was conducted to exam the migration and invasion-related proteins N-cadherin, MMP-2, MMP-9 and E-cadherin (*p < 0.05)

To clarify the role of LINC00461 in cell migration and invasion in breast cancer, si-NC and si-LINC00461 were transfected into MCF-7 and MDA-MB-157 cells. As indicated by qRT-PCR in Fig. 3a, LINC00461 was greatly decreased in cells transfected with si-LINC00461 relative to that in cells with si-NC (*p *< 0.05). Thereafter, Transwell was performed, finding the significantly reduced cell invasion and migration abilities in the si-LINC00461 group, as suggested by fewer invaded and migrated cells per field shown in Fig. 3b–e (*p *< 0.05). Moreover, migration and invasion-related proteins were test by Western blot. As shown in Fig. 3f, compared with the NC, N-cadherin, MMP-2 and MMP-9 were remarkably decreased in si-LINC00461 group, whereas E-cadherin was increased. Collectively, the findings demonstrated that silencing LINC00461 could inhibitory function on cell invasion and migration in breast cancer.

### LINC00461 silencing suppresses cell migration and invasion in breast cancer via promoting miR-144-3p

**Fig. 4:**
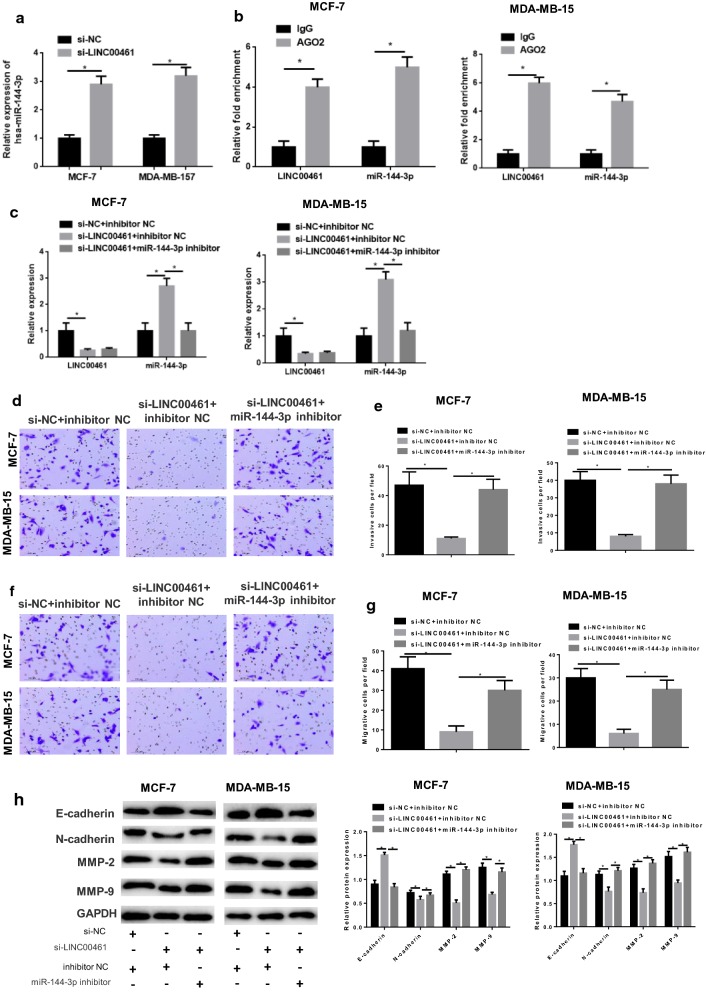
Silencing LINC00461 inhibits cell invasion and migration in breast cancer via promoting miR-144-3p. si-NC and si-LINC00461 were transfected into MCF-7 and MDA-MB-157 cells. **a** qRT-PCR was performed to detect the miR-144-3p level and **b** RIP was conducted to validate the combination of miR-144-3p and LINC00461. Then, miR-144-3p inhibitor and inhibitor NC were simultaneously transfected into cells, contributing to si-NC + inhibitor NC, si-LINC00461 + inhibitor NC and si-LINC00461 + miR-144-3p inhibitor groups. All cells were collected for **c** qRT-PCR to detect transfection efficiency, then subjected to Transwell to determine the cell **d**, **e** invasion and **f**, **g** migration abilities, as well as to **h** Western blot to test the invasion and migration-related proteins N-cadherin, MMP-2, MMP-9 and E-cadherin (*p < 0.05)

To gain more insight into the functional mechanism of LINC00461 in breast cancer, miR-144-3p expression was detected in cells via qRT-PCR, showing that miR-144-3p was significantly elevated in the si-LINC00461 group relative to that in the si-NC group (Fig. 4a, *p *< 0.05). In addition, RIP assay revealed that miR-144-3p could bind with LINC00461 (Fig. 4b, *p *< 0.05). These results elucidated that silencing LINC00461 could promote miR-144-3p expression.

Subsequently, we simultaneously inhibited miR-144-3p in the si-LINC00461-transfected cells and set a negative control group (si-NC + inhibitor NC). As indicated by qRT-PCR in Fig. 4c, LINC00461 was significantly down-regulated in si-LINC00461 + inhibitor NC transfected cells relative to the negative control, whereas miR-144-3p was up-regulated (both *p *< 0.05). Meanwhile, compared with the si-LINC00461 + inhibitor NC group, there was no much difference in LINC00461 expression with the si-LINC00461 + miR-144-3p inhibitor group, but miR-144-3p was remarkedly reduced (*p *< 0.05). In view of these, it suggested that LINC00461 could inhibit the expression of miR-144-3p. Then, cell biological behaviors were detected. Transwell revealed that cells transfected with si-LINC00461 + inhibitor NC had greatly decreased invasion and migration abilities relative to the cells in the si-NC + inhibitor NC group, which were both restored after miR-144-3p was concurrently inhibited (Fig. 4d–g, *p *< 0.05). Moreover, invasion and migration-associated factors were also determined. As shown in Fig. 4h, N-cadherin, MMP-2 and MMP-9 were considerably decreased whereas E-cadherin was increased in cells transfected with si-LINC00461 + inhibitor NC relative to those with si-NC + inhibitor NC, and such effects could be reversed when miR-144-3p was suppressed (*p *< 0.05). All above results collectively indicated that silencing LINC00461 was able to reduce cell migration and invasion abilities in breast cancer, while simultaneously down-regulating miR-144-3p could attenuate even reverse such inhibitory effect. To be more specific, LINC00461 silencing exerted its inhibitory role in cell migration and invasion in breast cancer via inducing miR-144-3p expression.

### miR-144-3p targets KPNA2

**Fig. 5:**
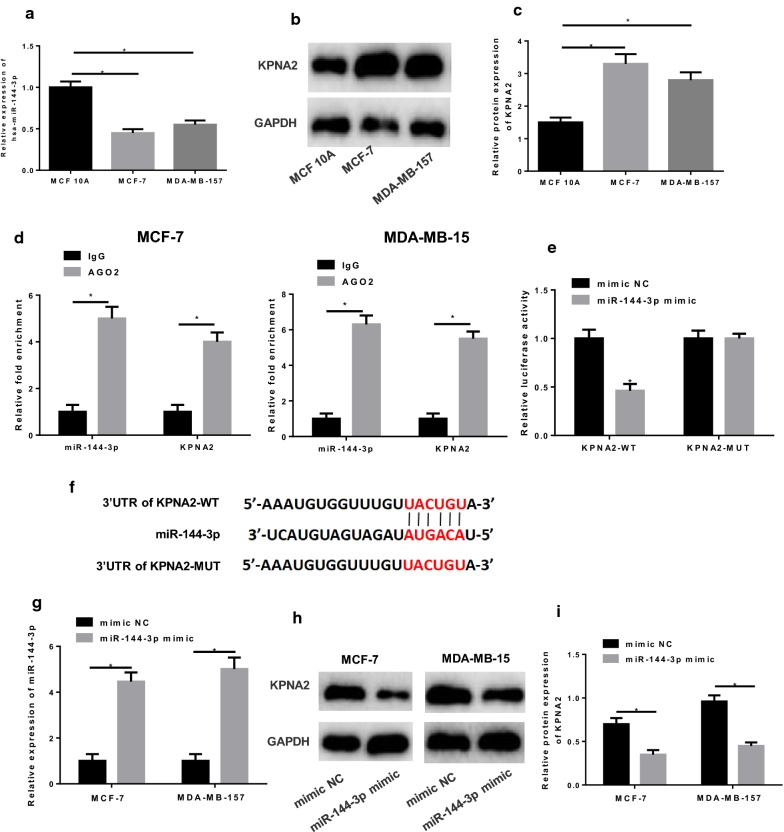
KPNA2 is a target of miR-144-3p. **a** qRT-PCR and **b**, **c** Western blot were conducted to determine miR-144-3p and KPNA2 levels in breast cancer cell lines MCF-7 and MDA-MB-157 and human normal breast epithelial cell line MCF 10A, respectively. **d** RIP and **e** dual-luciferase reporter assay were carried out for validation of the combination between miR-144-3p and KPNA2, and **f** their potential targeted binding sites were predicted by a bioinformatics database. Mimic NC and miR-144-3p mimic were transfected into cells, and the levels of **g** miR-144-3p and **h**, **i** KPNA2 were test again in cells (*p < 0.05)

qRT-PCR was performed to detect miR-144-3p in breast cancer cell lines MCF-7 and MDA-MB-157 and human normal breast epithelial cell line MCF 10A, finding the considerably lower expression of miR-144-3p in MCF-7 and MDA-MB-157 cells relative to that in MCF 10A cells (Fig. 5a, *p *< 0.05). Besides, Western blot was conducted to test KPNA2 and discovered that KPNA2 was greatly elevated in cancer cell lines in comparison with that in the normal epithelial cell line MCF 10A (Fig. 5b–c, *p *< 0.05).

Furthermore, RIP assay discovered that miR-144-3p could bind to KPNA2 (Fig. 5d, *p *< 0.05), and dual-luciferase reporter assay showed that the luciferase activity in miR-144-3p minic + KPNA2-WT transfected cells was greatly decreased relative to that in cells with mimic NC (Fig. 5e, *p *< 0.05), while there was no difference in KPNA2-MUT transfected cells (p > 0.05). These findings demonstrated that KPNA2 was a direct target of miR-144-3p. In addition, targeted binding sites of miR-144-3p on KPNA2 3′UTR were predicted using the bioinformatics database as revealed in Fig. 5f. To be more conceivable, qRT-PCR and Western blot were performed to test the expression of miR-144-3p and KPNA2 in miR-144-3p mimic transfected cells, respectively, finding the significantly increased miR-144-3p but decreased KPNA2 relative to the NC mimic group (Fig. 5g–i, *p *< 0.05). Overall, we concluded that miR-144-3p could targeted inhibit KPNA2.

### LINC00461 silencing is responsible for the inhibition of cell migration and invasion in breast cancer via the miR-144-3p/KPNA2 axis

**Fig. 6:**
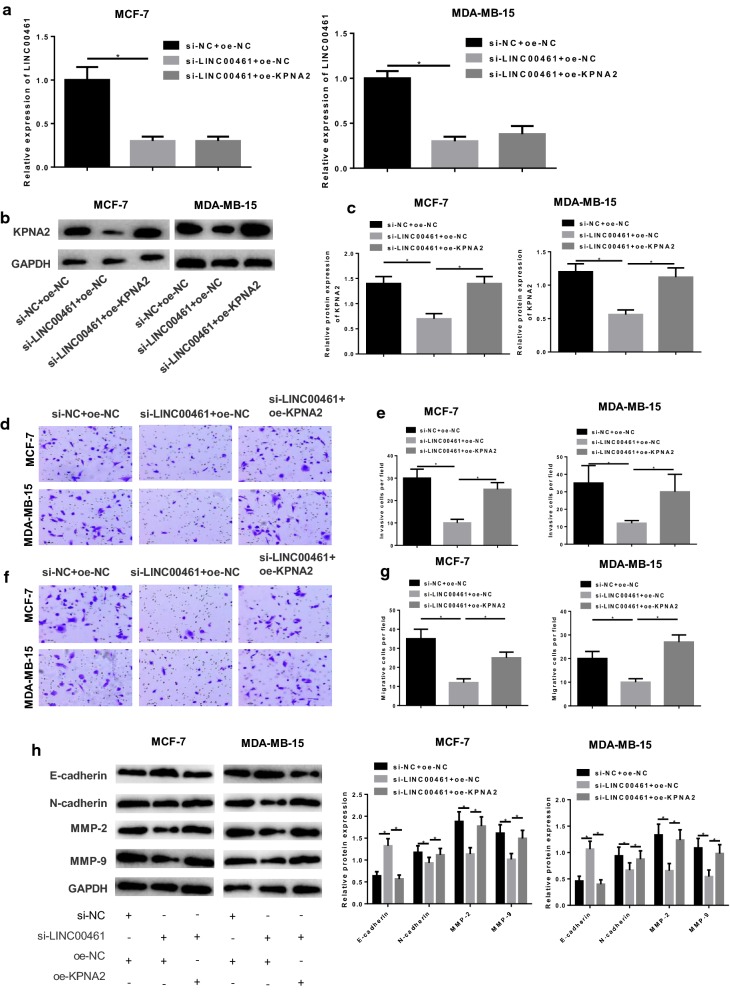
Silencing LINC00461 inhibits cell invasion and migration via the miR-144-3p/KPNA2 axis. si-NC + oe-NC, si-LINC00461 + oe-NC and si-LINC00461 + oe-KPNA2 were transfected into MCF-7 and MDA-MB-15 cells. **a** qRT-PCR and **b**, **c** Western blot were conducted to detect the levels of LINC00461 and KPNA2, respectively. Then the cells were harvested for Transwell for the assessment of cell **d**, **e** invasion and **f**, **g** migration. **h** Western blot was performed to assay the invasion and migration-related proteins (*p < 0.05)

As abovementioned, LINC00461 could bind with miR-144-3p and KPNA2 was a direct target of miR-144-3p. Hence, to achieve a better understanding of the regulatory mechanism of the LINC00461/miR-144-3p/KPNA2 axis underlying cell invasion and migration in breast cancer, we divided cells into si-NC + oe-NC, si-LINC00461 + oe-NC and si-LINC00461 + oe-KPNA2 groups. qRT-PCR and Western blot were conducted to detect the levels of LINC00461 and KPNA2 in each transfection group. As shown in Fig. 6a–c, LINC00461 was significantly reduced in si-LINC00461 + oe-NC group relative to the si-NC + oe-NC group (*p *< 0.05), yet there was no difference with the si-LINC00461 + oe-KPNA2 group (*p *> 0.05). Meanwhile, KPNA2 in si-LINC00461 + oe-NC transfected cells exhibited a greatly decreased expression in comparison with that in the si-NC + oe-NC group (*p *< 0.05), whereas in si-LINC00461 + oe-KPNA2 group KPNA2 was restored near to the si-NC + oe-NC group (*p *< 0.05). Then cell invasion and migration abilities were assayed via Transwell, indicating the reduced abilities in si-LINC00461 + oe-NC group relative to the si-NC + oe-NC group (*p *< 0.05), but the enhanced abilities in si-LINC00461 + oe-KPNA2 group compared with the si-LINC00461 + oe-NC group (Fig. 6d–g, *p *< 0.05). In addition, invasion and migration associated proteins were detected, presenting a much higher E-cadherin and lower N-cadherin, MMP-2 and MMP-9 in si-LINC00461 + oe-NC group relative to the si-NC + oe-NC group (*p *< 0.05), but opposite results were shown in si-LINC00461 + oe-KPNA2 group compared with the si-LINC00461 + oe-NC group (Fig. 6h, *p *< 0.05). Taken together, these results elucidated that silencing LINC00461 could inhibitory function on cell invasion and migration in breast cancer, and such effect could be reversed when KPNA2 was overexpressed. Thus, it could be concluded that silencing LINC00461 played an inhibitory role in cell invasion and migration in breast cancer via targeting miR-144-3p to suppressing KPNA2.

## Discussion

Breast cancer is one of the most common malignancies in women generally occurring in breast epithelial tissues and has been gradually developed as a threat to health even life, with a morbidity accounting for approximately 7–10% of total malignancies next to cervical carcinoma [19]. Nowadays, various therapeutic methods against breast cancer have been applied, but the efficacy remains very poor. Therefore, mining novel molecular targets for breast cancer treatment has become the present focus.

Increasing evidence has suggested that lncRNAs are implicated in the occurrence and development of diverse cancer types. For instance, LINC01433 is elevated in NSCLC and able to enhance the cell migration and invasion abilities [20]. LINC01420 overexpression can predict poor prognosis of patients with nasopharyngeal carcinoma, and LINC01420 knockout can suppress cell migration and invasion in vitro [21]. LINC01358 is found to be highly expressed in prostatic cancer, and LINC01358 knockout probably inhibitory functions on cell proliferation and migration [22]. Similarly, lncRNAs act as important players in breast cancer as well. Linc-ITGB1, for example, can potentiate cell migration and invasion [23], while low expression of LINC00152 is capable of suppressing cell viability, migration, invasion and reversing the chemoresistance [24].

In the present study, we downloaded the data of gene and miRNA expression quantification from the TCGA-BRCA dataset, and identified DEGs, DElncRNAs and DEmiRNAs, finding that LINC00461 exhibited a much higher expression in breast cancer. Given that LINC00416 has been reported to be able to promote cell invasion and migration in breast cancer [11], we made some further steps for validation. In addition, it has been revealed that LINC00461 can mediate cell invasion and migration in hepatocellular carcinoma via serving as a ceRNA that can competitively bind to miR-149-5p [10]. While in our study, we constructed a ceRNA network and discovered that LINC00461 might inhibit miR-144 expression, thereby regulating cell invasion and migration in breast cancer. miR-144 as reported is down-regulated in breast cancer, and overexpressing miR-144 can negatively function on cell invasion and migration through reducing CEP55 [25]. Meanwhile, miR-144 acts as a tumor suppressor in breast cancer by means of impeding ZEB1/2-induced EMT process [26]. This study confirmed that LINC00461 could interact with miR-144 and in turn targeted down-regulate its expression via the ceRNA network and RIP assay. This is the first study to indicate that LINC00461 acts as a ceRNA for miR-144.

Bioinformatics analysis suggested that LINC00461 probably inhibited miR-144 to promote KPNA2 expression, consequently mediating cell invasion and migration in breast cancer. Karyopherinα2 (KPNA2) has been validated to be elevated in various cancers, including cervical carcinoma [27], esophageal cancer [28], lung cancer [29] and hepatocellular carcinoma [30]. Besides, KPNA2 is overexpressed in human epithelial ovarian cancer tissues and cell lines, and intimately correlated with poor prognosis [31]. Our study described that KPNA2 was highly expressed in breast cancer and was identified to be a direct target of miR-144-3p by applying RIP and dual-luciferase reporter assay. As well, we discovered that LINC00461 silencing was implicated in the inhibition of cell invasion and migration, while overexpressing KPNA2 simultaneously could abrogate such negative effect. Thus, it could be concluded that silencing LINC00461 exerted its role in cell invasion and migration through sponging miR-144-3p to reduce KPNA2.

## Conclusion

In conclusion, our results further validated the high expression of LINC00461 in breast cancer tissues and cells, and confirmed that high expression of LINC00461 was able to predict poor prognosis. Besides, LINC00461 could mediate cell invasion and migration in breast cancer via the miR-144-3p/KPNA2 axis. Hence, the regulatory axis LINC00461/miR-144-3p/KPNA2 can be used as a promising therapeutic target in breast cancer treatment.

## Data Availability

The data used to support the findings of this study are available from the corresponding author upon request.
